# Cyclodextrins Modified/Coated Metal–Organic Frameworks

**DOI:** 10.3390/ma13061273

**Published:** 2020-03-11

**Authors:** Huacheng Zhang, Zhaona Liu, Jian Shen

**Affiliations:** 1 School of Chemical Engineering and Technology, Xi’an Jiaotong University, Xi’an 710049, Shanxi, China; 2 Medical School, Xi’an Peihua University, Xi’an 710125, Shanxi, China; zhaonaliu@peihua.edu.cn; 3 School of Chemistry, Chemical and Environmental Engineering, Weifang University, Weifang 261061, Shandong, China; shenjian@wfu.edu.cn

**Keywords:** metal–organic frameworks, cyclodextrins, modified, coated, hybrid materials, synthesis, applications

## Abstract

Recent progress about a novel organic–inorganic hybrid materials, namely cyclodextrins (CDs) modified/coated metal–organic frameworks (MOFs) is summarized by using a special categorization method focusing on the interactions between CDs and MOFs moieties, such as ligand–metal cations interactions, supramolecular interactions including host–guest interactions and hydrogen bonding, as well as covalent bonds. This review mainly focuses on the interactions between CDs and MOFs and the strategy of combining them together, diverse external stimuli responsiveness of CDs-modified/coated MOFs, as well as applications of these hybrid materials to drug delivery and release system, catalysis and detection materials. Additionally, due to the importance of investigating advanced chemical architectures and physiochemical properties of CDs-modified/coated MOFs, a separate section is involved in diverse characterization methods and instruments. Furthermore, this minireview also foresees future research directions in this rapidly developing field.

## 1. Introduction

Metal–organic frameworks (MOFs) are ordered porous solid crystalline coordination polymers [1], fabricated by polycomplexing linkers including carboxylate, phosphonates, sulfonates and imidazolates with easily tunable compositions such as various metal cations, and have wide applications in gas storage [2], separation and catalysis [1]. However, there are still some limitations and challenges for (1) preparing MOFs, e.g., usually requiring high reaction temperature and long reaction time [3], (2) tuning porosities of MOFs without affecting its stability, e.g., via external stimuli [4], as well as (3) applying MOFs to biomedicines or environmental areas in aqueous atmosphere, e.g., with poor colloidal stability [5]. Thus, moderate synthesis conditions, as well as additional functionalization of MOFs with biofriendly or stimuli-responsive moieties are highly desired, not only to control pore sizes and shapes [6,7], but also to provide the possibility of adjusting physiochemical properties of MOFs such as aqueous solubility and bioactivity [3].

**Scheme 1:**
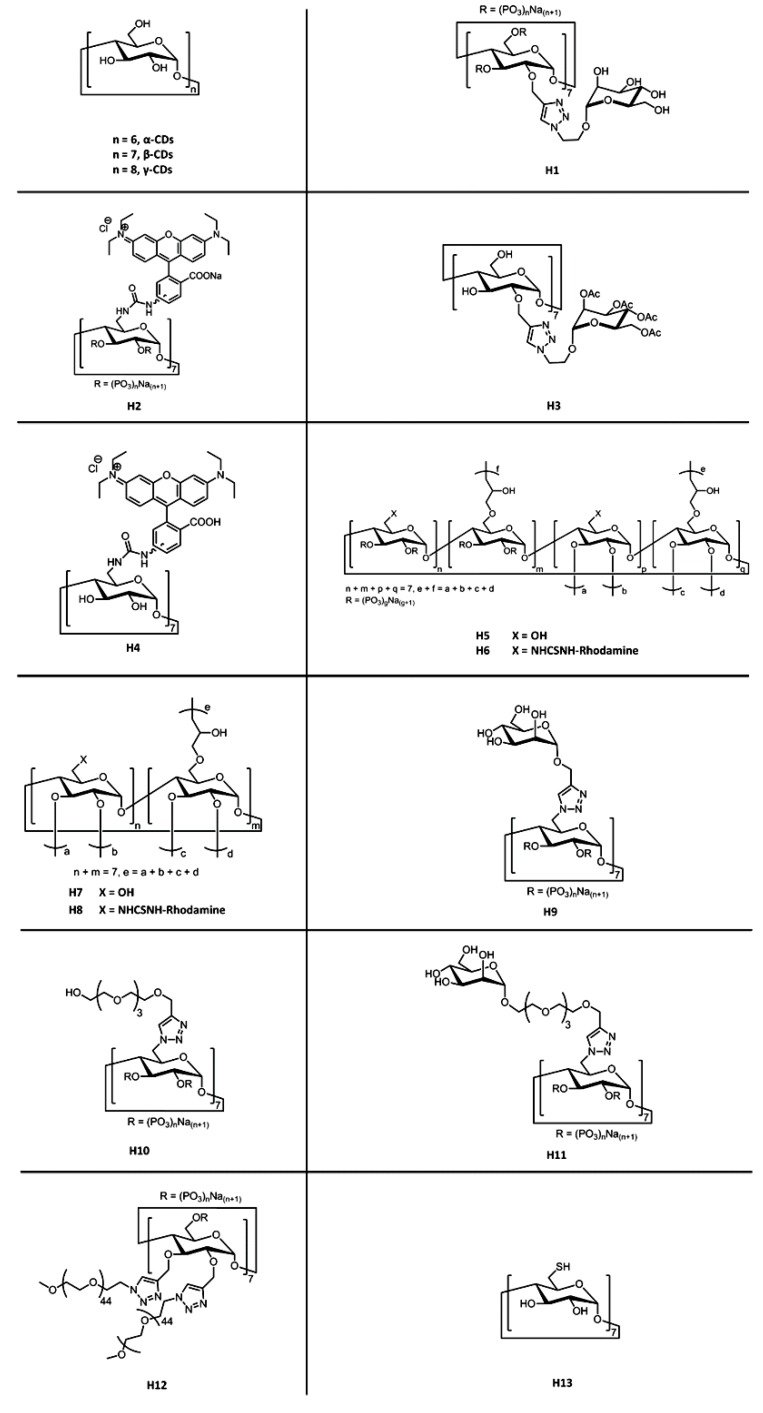
Chemical structural representation of cyclodextrins (CDs) and their derivatives H1–H13.

Cyclodextrins (CDs, Scheme 1) [8], composed by a macrocyclic structure of repeated glucose moieties via α-(1→4) glycosidic bonds, is a very good candidate to supplement the structure and improve the physiochemical properties of MOFs [9], and have been directly employed as the strut/ligand unit to complex with metal cations [10] in the fabrication of CDs-based MOFs [11,12,13,14] to, e.g., improve biocompatibility [15], add multiple porosities, as well as provide extra choices of introducing stimuli responsiveness through host–guest interactions [10]. Unfortunately, the water stability of CDs-MOFs is weakened due to its sensitivity towards water [12].

Very recently, a novel efficient strategy of introducing CDs into the MOFs materials appears via a more mild preparation condition without altering the capacity of MOFs for absorbing and storing guest molecules, i.e., the chemical architecture of general MOFs could be prepared first to enhance the thermal stability of integrated solid materials, and then biofriendly CDs could be employed into the MOFs materials via supramolecular interactions [16] such as metal cation–ligand interactions, host–guest interactions, hydrogen bonds and covalent bonds, leading to the fabrication of novel hybrid materials—CDs-coated/modified MOFs with improved aqueous solubility and biocompatibility.

**Scheme 2:**
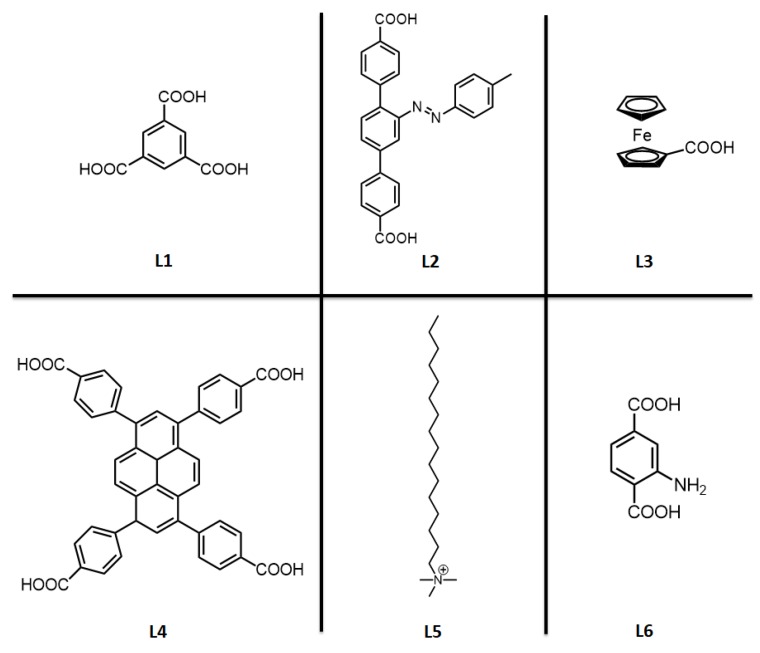
Chemical structural representation of selected struts of metal–organic frameworks (MOFs) and other significant molecules including L1–L6.

In this review, we briefly introduced the stable and highly porous CDs-modified/coated MOFs (Scheme 1 and Scheme 2, Table 1) [17,18,19,20,21,22,23,24,25,26,27,28]. Bridging CDs and MOFs chemistry is very challenging, and researchers with various backgrounds such as supramolecular chemistry, metal ligated materials and polymeric chemistry have done valuable contributions in this arising area. To attract more potential readers with diverse research interests, we plan to make a balance of different information during current research progress by presenting selected attractive contents in this review. For example, we plan not to show the synthesis details of those known precursors such as MOFs and modified CDs. A detailed description of the strategy of combining CDs and MOFs together, such as particular interactions and bonds between CDs and MOFs moieties, coupled with relative characterization techniques and data discussion would be envisaged to diverse researchers who might carry out the start-up work on this interfacial discipline. To avoid repeating the same instrument in different cases, we plan to employ a separate section about characterization methods and instruments to assist in understanding the process of confirming those advanced and complicated chemical architectures. Additionally, diverse external stimuli responsiveness of these hybrid materials will also be exhibited, because it is very important for applications in different practical fields including drug delivery and release system, catalysis and detection materials. Finally, the area of CDs-coated/modified MOFs is still under development, and has a lot of scientific and technical issues in current researches. We will try to target those issues and new challenges in the outlook section, and provide brief and primary suggestions for future work.

## 2. Characterization and Instruments

Multiple characterization methods and instruments are involved to investigate the organic–inorganic hybrid materials prepared by CDs-modified/coated MOFs, due to the possession of diverse compositions and various interactions in these advanced architectures. For example, the chemical structures of organic struts for preparing MOFs can be characterized by ^1^H NMR [17], some particular functional groups such as azobenzene moieties on precursors of MOFs can be determined by UV spectra [17,24], and phosphate groups can be checked by ^31^P NMR [23,27]. The employment of the same characterization methods and instruments in different processions of building advanced materials could always have different purposes, for example, powder X-ray diffraction (PXRD) patterns are adopted to compare with simulated data from the single X-ray crystal structure of MOFs [17,23,24,25,26,27], and also very direct proofs for confirming the crystallinity of CDs-modified MOFs [18,20,21]. In this section, we will discuss different characterization methods and instruments according to different purposes during diverse processions of the construction of CDs-modified/coated MOFs including preparing MOFs, studying interactions between CDs and MOFs, as well as confirming the integrated hybrid materials.

The composition, crystallinity, surface area and pore sizes of MOFs are very important for applying MOFs in different areas. Except for using ^1^H NMR to characterize the chemical structure of struts for MOFs, the composition of MOFs can also be quantitatively detected by ^1^H NMR via the digested MOFs samples [17]. The crystalline structures of MOFs can be directly revealed by single-crystal X-ray diffraction studies [17]. Nitrogen sorption measurement of MOFs can be used for checking their permanently porous nature by the Brunauer–Emmett–Teller (BET) analysis [17,20,21,22,23,24,26]. Additionally, the pore size distribution can be determined by density functional theory [17].

The interactions between CDs and MOFs can be monitored differently according to various driving forces and functional moieties. For example, UV spectra are used to check the absorption of azobenzene before and after being included by the cavity of CDs [17], and cyclic voltammetry (CV) is involved to confirm host–guest interactions where redox signals can be determined [19]. Isothermal titration calorimetry (ITC) can confirm the ligand–metal cations coordination [18,22,23], and particular information about interactions and bonds can be proved by ^1^H solid-state magic-angle spinning (MAS) NMR [18].

The loading of CDs into the MOFs systems can be determined by elemental analysis [18], spectrofluorimetry by using dyes-labeled CDs derivatives [18,23], Fourier transfer infrared [21,22,24,25,26] and thermogravimetric analysis (TGA) [21,22,24,25,29]. Confocal microscopy is useful for checking the load of dye-labeled CDs on the surface of MOFs [18]. The X-ray photoelectron spectroscopy (XPS) is employed to ascertain the location of loaded CDs within MOFs materials [18]. The crystallinity of CDs-coated MOFs can be further checked by transmission electron microscopy (TEM) [18,23,24,26,29] and scanning electronic microscopy (SEM) [20,21,22,24,25,26,29]. Sizes of CDs-coated MOFs can be determined by dynamic light scattering (DLS) [18,23,27].

## 3. Interactions and Bonds between CDs and MOFs

Combination of CDs and MOFs via metal cation–ligand interaction, supramolecular interactions such as host–guest interactions and hydrogen bonding, as well as covalent bonds can integrate advantages of both compositions together [17], for example, the structure of CDs provides a possibility of fabricating advanced hybrid materials via supramolecular interactions [20,22], and nano-sized space for storing and delivering special cargos such as hydrophobic guests [24]. In this part, we will introduce different CDs-modified/coated MOFs according to diverse interactions between compositions.

### 3.1. Metal Cation–Ligand Interaction

**Figure 1:**
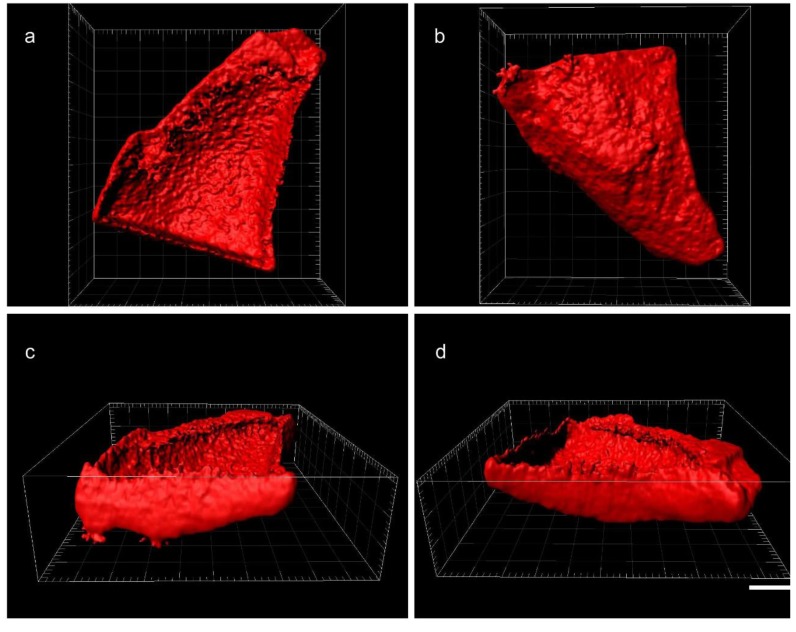
Reconstituted envelope of a MOF crystal coated with rhodamine-labeled **H1** and observed in a confocal microscope, which is an interesting characterization strategy for confirming porous frameworks. Views from top (**a**), bottom (**b**), left (**c**) and right (**d**). To enable visualization inside MOF, the signal from the first layer was removed. Scale bar = 10 µm. Reproduced from Reference [18] with permission from the Nature Publishing Group, copyright 2015.

The biodegradable and nontoxic Fe-MOFs—MIL-100(Fe) made by Fe^III^ octahedra trimers and 1,3,5-benzene tricarboxylate (**L1**, Scheme 2) possesses delimiting large (29 Å) and small (25 Å) mesoporous cages inside with openings as 8.6 Å and 5.6 Å, respectively, providing unsaturated Fe^III^ Lewis acid sites for binding specialized molecules such as the β-CDs bearing strong iron complexing groups—phosphates (**H1**, Scheme 1) [18]. It is found that **H1** could be firmly loaded on the surface of MIL-100(Fe) via phosphate–Fe^III^ interactions in aqueous solutions within 15 min (CDs loading amount = 13 wt %). Interestingly, the efficient coating procedure, i.e., simply mixing CDs with MOFs suspensions, did not affect the physiochemical properties of MOFs such as porosity, crystallinity, adsorption and release capacities (Figure 1). For example, the surface area of Fe-MOFs is maintained at 1350 ± 100 m^2^·g^−1^ (Langmuir) before and after the loading of **H1**, and the crystallinity of **H1**-coated Fe-MOFs is further confirmed by PXRD and TEM. Particularly, the introduction of **H1** on the outer surface of Fe-MOFs provides active sites for further anchoring biocompatible and targeting moieties, e.g., adamantyl-grafted poly(ethylene glycol) (PEG) by supramolecular methods.

**Figure 2:**
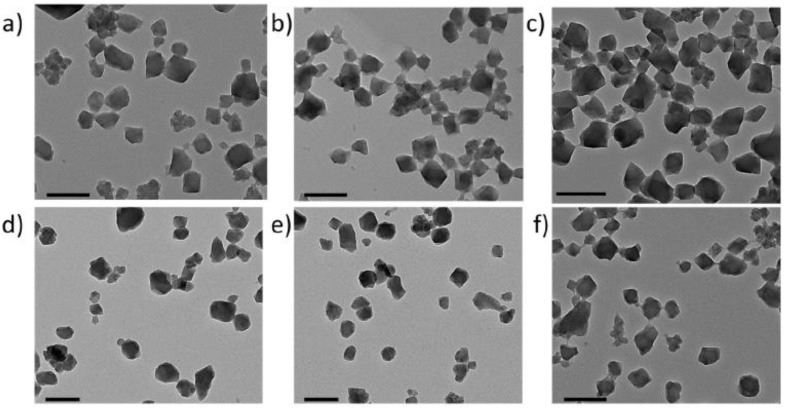
TEM images of MIL-100(Fe) before and after modification with β-CDs derivatives: (**a**) unmodified MIL-100(Fe); (**b**) β-CD phosphate sodium salt purchased [27] modified MIL-100(Fe); (**c**) **H9** modified MIL-100(Fe); (**d**) **H10** modified MIL-100(Fe); (**e**) **H11** modified MIL-100(Fe); (**f**) **H12** modified MIL-100(Fe). Scale bar = 200 nm. These pictures indicate that after the introduction of CDs, the obtained organic–inorganic hybrid materials kept similar sizes and the rigid architectures were not affected. Reproduced from Reference [27] with permission from MDPI, copyright 2019.

In another example, several β-CDs-based monomers such as **H1** and **H2** (β-CDs precursors as **H3** and **H4**, Scheme 1) and polymers such as **H5** and **H6** (polymeric β-CDs precursors as **H7** and **H8**, Scheme 1) are appended with mannose or rhodamine via copper-catalyzed azide-alkyne cycloaddition reaction or substitution reaction, and then randomly phosphorylated. Then, they are also used for coating on the outer surface of MIL-100(Fe) in water [23]. It is further found that the coating stability of β-CDs is directly related to the density of grafted phosphate moieties, multivalent effect by, e.g., persubstituted discrete β-CDs derivatives, as well as additional factors involved in polymers including steric hindrance, spatial distribution, induced-fit effects towards the Fe^III^ coordination centers, and the possibility of cross-linking between different nanoparticles. The coating of β-CDs does not affect either the morphology of MIL-100(Fe) or cargo release kinetics. Additionally, by using the same synthetic strategy [23,27], tetraethylene glycol and mannose residues are able to functionalize β-CDs phosphates on the primary face (**H9**–**H11**, Scheme 1), while polyethylene glycol can functionalize β-CDs phosphates on the secondary face (**H12**, Scheme 1) [27]. All these biocompatible β-CDs derivatives further anchor to the surface of MIL-100(Fe) for producing organic–inorganic hybrid materials (Figure 2).

Except for MIL-100(Fe), other MOFs can also coordinate with CDs to afford the hybrid materials. For example, the MOF-235/β-CDs hybrid material is prepared through possible coordination interaction between the hydroxyl groups on β-CDs and the unsaturated Fe^III^ cations in MOF-235 [25]. It is found that the chemiluminescence signal of the hybrid material gradually enhanced as the concentration of β-CDs increased, indicating that the introduction of β-CDs is important for improving the catalytic activity of MOF-235.

### 3.2. Host–Guest Interaction

**Figure 3:**
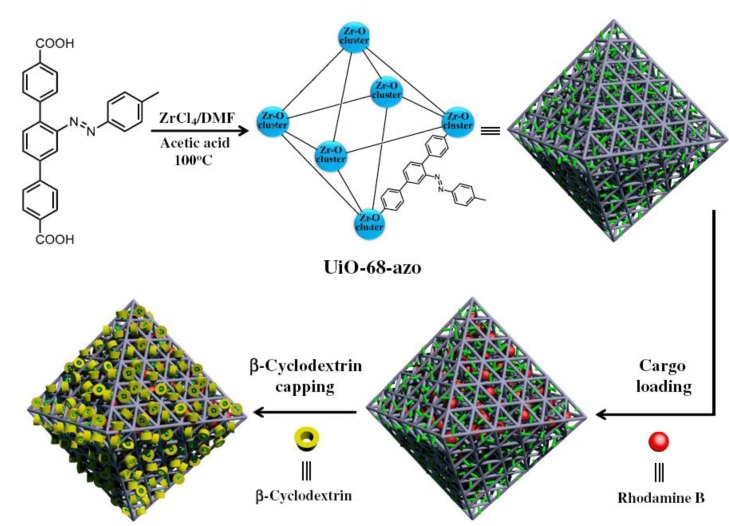
Schematic illustration of UiO-68-azo, as well as rhodamine B-loaded, β-CD-capped UiO-68-azo. The *trans*-azobenzene is colored in green. Reproduced from Reference [17] with permission from the American Association for the Advancement of Science, copyright 2016.

The water-stable robust zirconium MOF (Zr-MOF) bearing photoresponsive azobenzene groups, UiO-68-azo [17] was prepared first by using 2’-p-tolyldiazenyl-1,1’:4,4”-dicarboxylic acid (**L2**, Scheme 2), ZrCl_4_, acetic acid and DMF at 100 °C. Due to the possession of porous architectures with a high surface area of 2900 m^2^ g^−1^ and the average pore size centered at 1.4 nm, Zr-MOF bearing UiO-68-azo can be used for storing cargo such as rhodamine B in water. The β-CDs is further mixed with cargo-loaded Zr-MOFs, and the cargo-loaded Zr-MOF can be capped to form a mechanized MOF through the host–guest interaction between β-CDs and the *trans*-azobenzene moieties beside porous openings on the outer surface of crystalline MOFs. Thus, the pores on the outer surface are sealed, and the spontaneous release of cargo trapped inside Zr-MOF is further prevented (Figure 3). The conformational transformation of azobenzene moiety between *trans*-/*cis*-forms exposed under UV/vis light further results in the association and disassociation with the cavity of β-CDs, respectively. Thus, upon the trigger of UV irradiation, the β-CDs capped Zr-MOFs can release cargo inside MOFs without premature release [17]. Furthermore, the addition of competitive agents such as amantadine can also cause a similar controlled release of cargo.

In another example, to control electron transfer rates within MOFs architectures or between MOFs components and solution species, β-CDs is employed for modulating the kinetics of charge-hopping between ferrocene and ferrocenium sites in **L3** (Scheme 2) anchored MOFs based on presynthesized NU-1000 by **L4** (Scheme 2) and Zr^IV^. The modulation process is achieved via the different host–guest interactions of β-CDs with ferrocene/ferrocenium moieties [19], i.e., in polar solvents, the host–guest association constant (*K_a_*) of β-CDs/ferrocene is higher than 10^3^ mol L^−1^, while that of β-CDs/ferrocenium is less than 20 mol L^−1^. It is further found that for this specific example of β-CDs and NU-1000-channel-tethered ferrocene, the microscopic rate constant (*K_hop_*) for charge transfer between neighboring redox is able to be modulated by up to 30-fold.

### 3.3. Hydrogen Bonding

A lot of supported MOF material has been explored for improving application effectiveness of MOFs by using the method borrowed from supramolecular chemistry. For example, the 2/1 host–guest complex between β-CDs and cetyltrimethylammonium bromide (CTAB, **L5**, Scheme 2) could be effectively loaded onto the outer crystalline surface of MOFs, UiO-66-NH_2_ synthesized by Zr^IV^ and 2-aminoterephthalic acid (**L6**, Scheme 2), via hydrogen bonds, and acts as surfactant assembly agents [20] to promote the assembly of UiO-66-NH_2_ crystals on nonwoven polypropylene fibrous mats at ambient temperature. It could simultaneously impede solution phase agglomeration of MOFs. Interestingly, if the polypropylene is preconditioned by using conformal metal oxide thin films such as Al_2_O_3_, TiO_2_ or ZnO through atomic layer deposition (ALD), the hydrophilic metal oxide outer surface has the capacity of assisting in loading more MOFs (up to 40 wt %), and improving the BET surface area (200 m^2^ g^−1^_(MOF+fiber)_) of polypropylene fibers.

In another example, the 2/1 host–guest complex between β-CDs and **L5** was further used as the surfactant agents for assisting the assembly of NH_2_-MIL-101(Cr), which has large specific surface area and high thermal stability, on the surface of dopamine modified polyimide fibers, leading to the overall surface area of MOFs modified fibers reaching 800 m^2^ g^−1^ [22].

**Figure 4:**
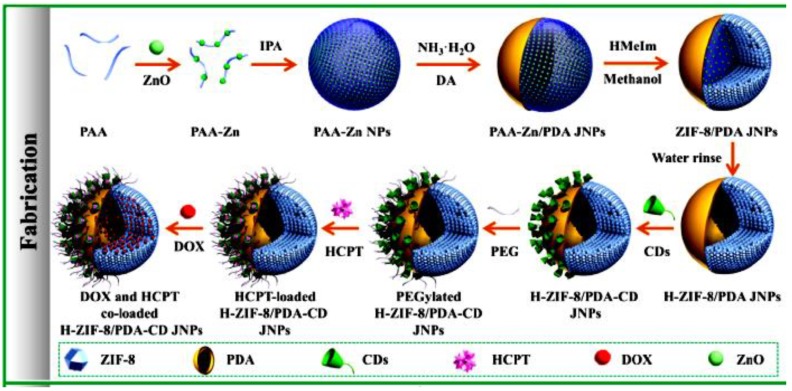
Schematic illustration of fabricating the H-ZIF-8/PDA-CD JNPs. The starting synthetic materials are poly(acrylic acid) (PAA), isopropyl alcohol (IPA), 2-methylimidazole (HMeIm) and dopamine (DA). The PEG was later employed for improving the stability of JNPs. Reproduced from Reference [28] with permission from Elsevier B. V. Copyright 2019.

Except for using CDs and its host–guest inclusions as surfactant assembly agents, CDs can also perform as extra storage devices. For example, the outer surface of MOFs, Fe-MIL-88B-NH_2_ prepared by **L6** and Fe^III^, could be further modified by β-CDs via hydrogen bonds [21]. Both the porosities of Fe-MIL-88B-NH_2_ and the cavities of loaded β-CDs could store drug models such as alendronate. In a very special example, heptakis-(6-mercapto-6-deoxy)-β-CDs (**H13**, Scheme 1) is connected with spherical zeolitic imidazolate framework-8 (ZIF-8) via the “bridge” reagent, polydopamine (PDA) for the fabrication of Janus nanoparticles (JNPs) with one side as β-CDs@PDA and the other side as H-ZIF-8 (Figure 4), which can recognize hydrophobic drugs such as 10-hydroxycamptothecin (HCPT) through the cavity of β-CDs, and hydrophilic drugs such as doxorubicin (DOX) through the pores inside ZIF-8 (Table 1) [28].

### 3.4. Covalent Modifications

Except for combining CDs with MOFs via supramolecular interactions, general covalent bonds are also involved to bridge them together [29]. For example, the hybrid material of magnetic zinc-metal–organic framework (M-MOF made by **L6** and Zn^II^)/β-CDs is prepared by creating M-MOF layers on the surface of a Fe_3_O_4_-graphene oxide (GO) nanocomposites in order to improve the water-resistance of MOFs, and covalently bonding them with carboxyl-β-CDs purchased without showing the correct structures in order to enhancing selective adsorption capacities for targeted cargos, resulting in superparamagnetism and a large surface area [26].

In another particular example of β-CDs mixing with mercapto-functionalized Fe_3_O_4_@SiO_2_@MIL-100(Fe) [24,29], the hydroxyl groups on CDs, the grafting agent allyl glycidyl ether, the initiator agent—2,2′-azobis(2-methylpropionitrile)—and mercapto-functionalized MIL-100(Fe) presented a great possibility to afford covalent bonds, combining organic and inorganic materials together [24]. The absorption band of β-CDs at around 1035 cm^−1^ observed by FT-IR spectra in the final hybrid materials, the changed pattern by XRD and the changed morphology of materials shown in SEM images after the addition of β-CDs, reveal that the modified Fe_3_O_4_@SiO_2_@MIL-100(Fe) has been coated by β-CD.

## 4. Applications

### 4.1. Drug Delivery and Release System

CDs-modified MOFs provide an opportunity of combining diverse functions for the application in biomedicines such as improving biocompatibility and solubility of delivery systems, introducing particular functional groups for recognizing specific targeting receptors, escaping immune system, delivering various drugs and drug models, release drugs according to external stimuli as well as delaying the release for different requirements of medical treatments [18]. Different roles of CDs and MOFs in the hybrid materials (Table 1) contribute a lot to improving the functions of traditional bioactive materials, as well as drug delivery and release systems.

**Figure 5:**
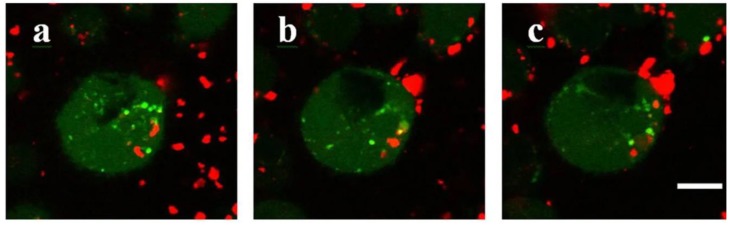
Observations by confocal microscopy to evaluate the interaction between J774 macrophase and CDs-coated MOFs, indicating that hybrid materials have penetrated into macrophase and affected its functions. Cells were stained in green with calcein, the cell nucleus is in black, and the rhodamine-B-labeled hybrid materials exhibited red signals. The differences of images (**a**–**c**) of the same cells caused by three different heights above the glass slide including 2.3, 4.9 and 7.6 µm, respectively. Scale bar = 5 µm. Reproduced from Reference [18] with permission from the Nature Publishing Group, copyright 2015.

The introduction of CDs can improve the biocompatibility and solubility of unmodified MOFs in H_2_O, and CDs-modified MOFs is able to be used for specific targeting, due to the possession of recognition ability on the outer surface of MOFs and the escape from the immune system. For example, in order to evade the immune system and to add extra molecular recognition ability, tetraethylene glycol, PEG and mannose moieties can functionalize CDs first, and then modified CDs could further combine with MOFs, such as loading CDs on the outer surface of MIL-100(Fe) via the phosphate–Fe^III^ bonding [18,27] and leading to a significant decrease of macrophage uptake (Figure 5). Particularly, the tetraethylene glycol-bearing β-CDs phosphate (**H11**) modified MOFs exhibit remarkably enhanced binding affinity towards the specific mannose receptor, i.e., Concanavalin A, due to the possession of mannose moieties, reduced macrophage internalization and multivalent effects based on the scaffold of β-CDs.

CDs-modified MOFs can be used for delivering and releasing drugs. In the first step, CDs mainly perform as a cap to stop the release of loaded drugs, for example, tetraethylene glycol functionalized β-CDs (**H10**) can further be coated on the outer surface of DOX-loaded MIL-100(Fe) without affecting the payload of the nanocomposite [27]. In the second step, removing CDs from hybrid materials will lead to the release of loaded drugs, and different CDs coated on the surface of inorganic materials have different performances, for example, the effect of releasing the tritium-labeled antiretroviral drug, AZT-PT by CDs-coated MOFs is estimated by scintillation counting [23], and reveals that polymeric β-CDs (such as **H5**) modified MOFs afford a better control of drug release over monomeric β-CDs (**H1** and **H2**) coated ones, due to the presence of nonmodified CDs moieties in the structure and interacting with drugs. Although it is known that AZT has interactions with native and primary face-modified β-CDs, polymeric β-CDs-modified MOFs can increase drug retention in 13%.

**Figure 6:**
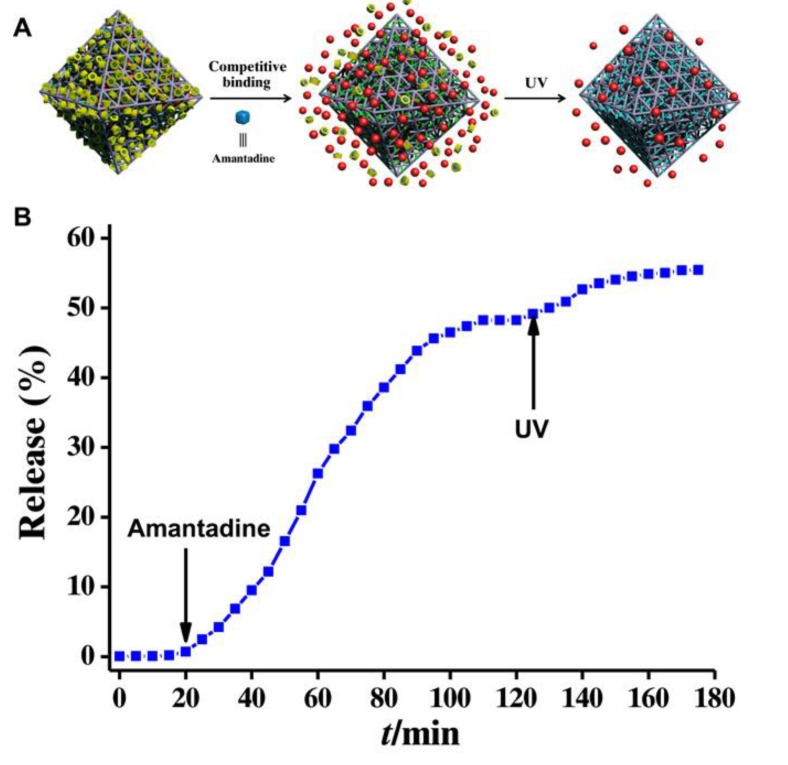
(**a**) Schematic illustration of the controlled release system upon the addition of a competitive guest, amantadine, as well as exposure under UV irradiation. The *trans*-azobenzene and its *cis*-isomer are colored in green and cyan, respectively. (**b**) Changing points in the release profile of CDs capped UiO-68-azo-bearing Zr-MOFs. Reproduced from Reference [17] with permission from the American Association for the Advancement of Science, copyright 2016.

Particularly, the external stimuli-responsive release of drugs can be achieved by the employment of CDs-coated MOFs. For example, external stimuli-responsive molecules such as azobenzene can be introduced into the hybrid materials such as the water-stable, biocompatible and degradable Zr-MOF, then act as the switch tool to associate/disassociate with coated β-CDs in accordance with different light irradiation conditions and, finally, control the performance of thus obtained integrated hybrid organic–inorganic materials, which have already stored drug models such as rhodamine B [17]. Additionally, the “gatekeeper”, β-CDs, can also be removed to release cargos inside the UiO-68-azo-bearing Zr-MOFs upon the addition of competitive guests, e.g., the drug for Parkinson’s disease, amantadine [17]. Thus, this kind of β-CDs-coated Zr-MOFs might be used as a platform for on-command drug delivery systems (Figure 6).

**Figure 7:**
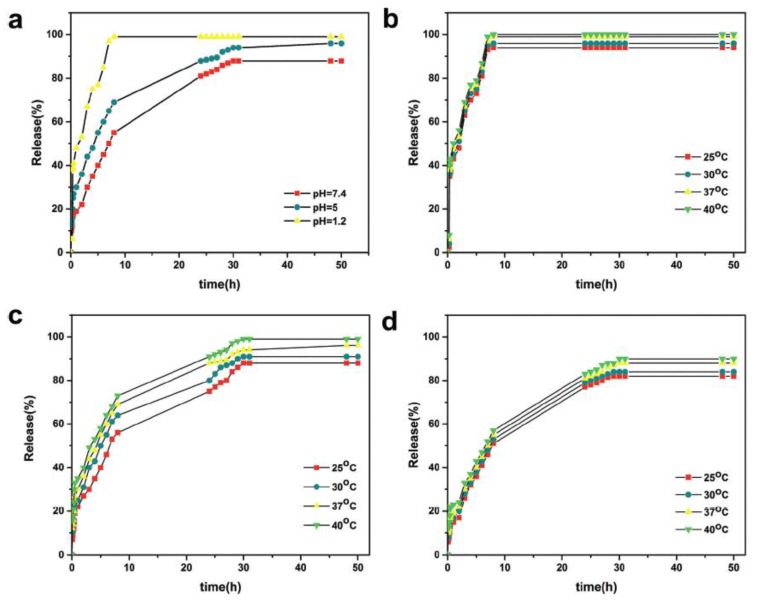
(**a**) The release of cephalexin at different pH at T = 37 °C, (**b**) the effect of temperature on the release of cephalexin at pH = 1.2, (**c**) the effect of temperature on the release of cephalexin at pH = 7 and (**d**) the effect of temperature on the release of cephalexin at pH = 5. Reproduced from Reference [24] from the Royal Society of Chemistry, copyright 2018.

Additionally, the organic–inorganic hybrid material, β-CDs/Fe_3_O_4_@SiO_2_@MIL-100(Fe) [24], can be used as a pH-responsive drug delivery system due to the possession of pH-sensitive β-CDs, which is unstable and easily hydrolyzed at lower pH solutions. It is found that the drug model, cephalexin is released by this system slower under physiological conditions than the acidic buffer solution, indicating that the release happens in a controlled manner. Furthermore, it is observed that increasing temperature enhanced the drug release percentage, due to the shrinking of temperature-sensitive polymers inside hybrid materials (Figure 7). However, the coating of the organic layer onto the surface of Fe_3_O_4_@SiO_2_@MIL-100(Fe) results in a decrease in magnetic responsivity, and the magnetic responsiveness of this hybrid material was not explored directly upon releasing drugs.

CDs-modified MOFs can perform as efficient and robust drug delivery systems comprising potential for slow release. For example, β-CDs-modified Fe-MIL-88B-NH_2_ [21] could store alendronate through both the pores of MOFs and the cavities of loaded β-CDs, increasing the drug loading amount. Interestingly, after loading alendronate, the β-CDs-modified Fe-MIL-88B-NH_2_ can be encapsulated with hydroxyapatite, which is the main component of teeth texture and can further lower the drug release rate with bone-like structures by decreasing the demand dosage. It is still very curious about the role of CDs in this system, for example, it looks like both CDs and MOFs have the same roles in the construction of drug delivery systems here, and there is no control experiment about the delivery system without functionalizing CDs.

### 4.2. Catalysis and Detection Materials

The employment of CDs is a very good control strategy for tuning MOFs to apply in the catalysis area. For example, with the assistance of host–guest complex between β-CDs and **L5** as assembly agents, UiO-66-NH_2_ loaded polypropylene performs well in catalytic degradation of dimethyl 4-nitrophenyl phosphate, a chemical warfare agent simulant, with a half-life of less than 5 min [20].

**Figure 8:**
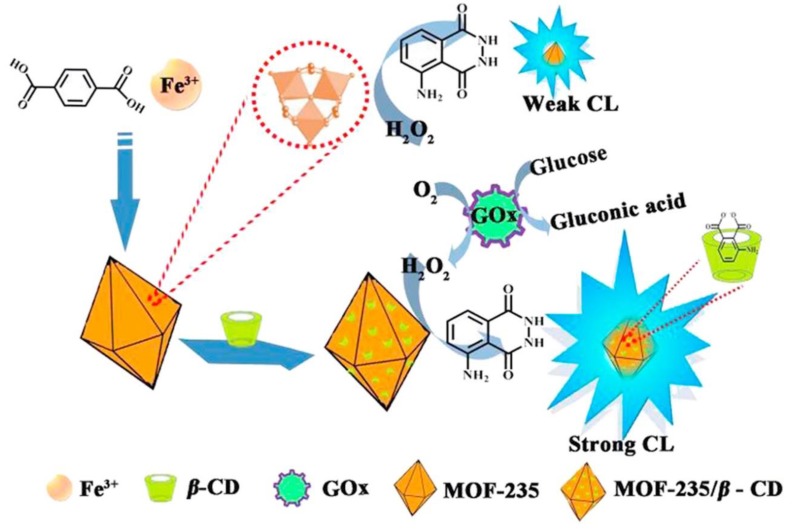
Proposed chemiluminescence (CL) mechanism of the luminol-H_2_O_2_ system by MOF-235/β-CDs hybrid materials. MOF-235 is generated by the coordination reaction between Fe^III^ and terephthalic acid (top left), and further functionalized with β-CD. The intermediate of luminol is supposed to be hosted by the cavity of β-CD (right) during the oxidation reaction of glucose with glucose oxidase (GOx). Reproduced from Reference [25] with permission from Elsevier B.V. Copyright 2018.

Additionally, the CDs-modified MOFs hybrid materials also reveal good catalytic behavior, due to the possession of the large surface area of MOFs as well as the synergistic effect between MOFs and CDs, for example, the nanocomposite, MOF-235/β-CDs has catalytic activity for the hydrogen peroxide-luminol system, and can enhance the chemiluminescence response with more than 30-fold in comparison with that of hydrogen peroxide-luminol system (Figure 8) [25]. Thus, the catalytical performance of MOF-235/β-CDs could be applied to sensitively detect H_2_O_2_ and glucose. It is further found that the limits of detection (LOD) for H_2_O_2_ and glucose in serum samples by MOF-235/β-CDs hybrid materials are 0.5 and 1.0 × 10^−9^ mol L^−1^, respectively.

Except for the above example for applying to detection fields, CDs-modified MOFs could further be used for extraction and separation. For example, the hybrid material M-MOF/β-CDs could perform as the solid phase extraction material and extract prochloraz and triazole fungicides from tomato and lettuce vegetables. As determined by HPLC–MS/MS, the LOD for those fungicides are 0.25–1.0 × 10^−6^ g L^−1^ at a signal-to-noise ratio of three with spiked recoveries of 74.13%–119.83% [26], indicating that analyte detection was not affected by differences in the standard curve diluent and experimental sample matrix.

## 5. Summary and Outlook 

In this review, we briefly introduced the organic–inorganic hybrid materials, namely CDs-modified/coated MOFs. Although there were a lot of categorization methods to discuss this topic such as different types of MOFs and modified/native CDs, as well as different roles of various compositions of the hybrid materials (Table 1), we mainly employed the categorization focusing on the interactions between CDs and MOFs moieties including ligand–metal cations interactions, supramolecular interactions such as host–guest interactions and hydrogen bonding, as well as covalent bonds. We also introduced diverse characterization methods and instruments, which are very important for studying and confirming these advanced and complicated materials. In this review, we also discussed the synthesis and preparation method, and the diverse external stimuli responsiveness of these hybrid materials, which affects the further applications a lot. Additionally, we have a separate section focusing on the applications of CDs-modified/coated MOFs including drug delivery and release system, as well as catalysis and detection materials.

A lot of perspective work in this area is still attractive for researchers in synthesis and material sciences, for example, (1) different sized CDs such as α- and γ-CDs should be considered for the construction of novel CDs-modified/coated MOFs, since they have different sizes and physiochemical properties compared to β-CDs [8]. (2) Functional groups with *N*-donors [2] should be considered for modifying CDs, which will greatly enrich current CDs materials and bring stronger interactions with MOFs moieties for building more stable hybrid materials. (3) some current researches need to be further explored, for example, some functional moieties such as magnetic materials [24] involved in the current advanced hybrid structures have not yet been fully used for practical applications. It will be very interesting whether CDs-based amphiphiles could directly perform as surfactant assembly agents or not [20]. Additionally, interactions between CDs and MOFs such as phosphate-Fe^III^ bonding [18,27] might weaken the stability of integrating hybrid materials, and the kinetic studies about thermal stability should be explored further to monitor the possible physiochemical changes accordingly. (4) The different abilities upon recognizing various guest molecules such as hydrophobic and hydrophilic ones by the cavities of CDs and porous structures of MOFs have not been fully explored [30], for example, up to now, there are no reports about using both CDs and MOFs in the hybrid materials to simultaneously delivery diverse cargos [28]. (5) The difference in porous sizes between MOFs and CDs has already been applied by directly mixing them together, e.g., for selective chiral separation by capillary electrochromatography [31], but it has not yet been explored in CDs-modified/coated MOFs.

## Figures and Tables

**Table 1:** The various compositions of CDs-modified/coated MOFs indicating different roles of CDs and MOFs, the supramolecular interactions and covalent bonds between them, as well as possible external stimuli responsiveness to control the organic–inorganic hybrid materials for future applications.

CDs	Role of CDs	MOFs	Role of MOFs	Interactions	Stimuli Responsiveness	Ref.
β-CDs	Cape to stop releasing cargo	UiO-68-azo Zr-MOF	Storing cargo in water	Azobenzene–β-CDs host–guest interactions	UV irradiation/competitive agent	[17]
H1	Improving poor colloidal stability, controlling the release of cargos, providing active recognition sites	MIL-100(Fe)	Storing cargo in water	Phosphate–Fe^III^ interactions	-	[18]
β-CDs	Modulate rates of charge transport in MOFs films	NU-1000	Charge transport	Ferrocene–β-CDs host–guest interactions	Redox	[19]
β-CDs⸧L5 complex	Surfactant assembly agents	UiO-66-NH_2_	Catalyst for degradation of chemical warfare agents	Hydrogen bonds	-	[20]
β-CDs	Storing drug	Fe-MIL-88B-NH_2_	Storing the same drug	Hydrogen bonds	-	[21]
β-CDs⸧L5 complex	Surfactant assembly agents	NH_2_-MIL-101(Cr)	Filtration	Hydrogen bonds	-	[22]
H1, H2, H5, H6	Increasing the solubility of hydrophobic cargos, improving poor colloidal stability, controlling the release of cargos, providing active recognition sites	MIL-100(Fe)	Storing cargo in water	Phosphate–Fe^III^ interactions	-	[23]
β-CDs	pH-responsive moiety	MIL-100(Fe)	Storing drug	Possible covalent bonds	pH/temperature	[24]
β-CDs	Enhancing the catalytic performance of MOFs	MOF-235	Catalysis	Hydroxyl–Fe^III^ interactions	-	[25]
carboxyl-β-CDs	Enhancing selective adsorption capacities for guests	M-MOF (Zn)	Enlarging surface area	Covalent bonds	-	[26]
H9–H12	Assisting in escaping immune system and enhancing biocompatibility	MIL-100(Fe)	Storing drug	Phosphate–Fe^III^ interactions	-	[27]
H13	Loading hydrophobic drugs	ZIF-8	Responsive active sites/storing hydrophilic drugs	Polydopamine acting as the bridge	-	[28]

## Abbreviation

ALD	Atomic layer deposition
BET	Brunauer–Emmett–Teller
CTAB	Cetyltrimethylammonium bromide
CDs	Cyclodextrins
CL	Chemiluminescence
CV	Cyclic voltammetry
DA	Dopamine
DOX	Doxorubicin
DLS	Dynamic light scattering
GO	Graphene oxide
HCPT	10-hydroxycamptothecin
HMeIm	2-methylimidazole
IPA	Isopropyl alcohol
ITC	Isothermal titration calorimetry
JNPs	Janus nanoparticles
*K_a_*	Association constant
*K_hop_*	Microscopic rate constant
LOD	Limits of detection
MAS	Magic-angle spinning
MOFs	Metal–organic frameworks
PAA	Poly(acrylic acid)
PEG	Poly(ethylene glycol)
PDA	Polydopamine
PXRD	Powder X-ray diffraction
SEM	Scanning electronic microscopy
TGA	Thermogravimetric analysis
TEM	Transmission electron microscopy
XPS	X-ray photoelectron spectroscopy
ZIF	Zeolitic imidazolate framework
